# Personal and Professional Factors Associated With Work-Life Integration Among US Physicians

**DOI:** 10.1001/jamanetworkopen.2021.11575

**Published:** 2021-05-27

**Authors:** Daniel S. Tawfik, Tait D. Shanafelt, Liselotte N. Dyrbye, Christine A. Sinsky, Colin P. West, Alexis S. Davis, Felice Su, Kathryn C. Adair, Mickey T. Trockel, Jochen Profit, J. Bryan Sexton

**Affiliations:** 1 Division of Pediatric Critical Care Medicine, Department of Pediatrics, Stanford University School of Medicine, Stanford, California; 2 Department of Medicine, Stanford University School of Medicine, Stanford, California; 3 Department of Internal Medicine, Mayo Clinic, Rochester, Minnesota; 4 American Medical Association, Chicago, Illinois; 5 Division of Neonatology, Department of Pediatrics, Stanford University School of Medicine, Stanford, California; 6 Duke Center for Healthcare Safety and Quality, Duke University Health System, Durham, North Carolina; 7 Department of Psychiatry, Stanford University School of Medicine, Stanford, California; 8 Perinatal Epidemiology and Health Outcomes Research Unit, Division of Neonatology, Department of Pediatrics, Stanford University School of Medicine, Stanford, California; 9 California Perinatal Quality Care Collaborative, Palo Alto; 10 Department of Psychiatry, Duke University School of Medicine, Duke University Health System, Durham, North Carolina

## Abstract

**Importance:**

Poor work-life integration (WLI) occurs when career and personal responsibilities come in conflict and may contribute to the ongoing high rates of physician burnout. The characteristics associated with WLI are poorly understood.

**Objective:**

To identify personal and professional factors associated with WLI in physicians and identify factors that modify the association between gender and WLI.

**Design, Setting, and Participants:**

This cross-sectional study was based on electronic and paper surveys administered October 2017 to March 2018 at private, academic, military, and veteran’s practices across the US. It used a population-based sample of US physicians across all medical specialties. Data analysis was performed from November 2019 to July 2020.

**Main Outcomes and Measures:**

WLI was assessed using an 8-item scale (0-100 point scale, with higher scores indicating favorable WLI), alongside personal and professional factors. Multivariable linear regressions evaluated independent associations with WLI as well as factors that modify the association between gender and WLI.

**Results:**

Of 5197 physicians completing surveys, 4370 provided complete responses. Of the physicians who provided complete responses, 2719 were men, 3491 were White/Caucasian (80.8%), 3560 were married (82.4%), and the mean (SD) age was 52.3 (12.0) years. The mean (SD) WLI score was 55 (23). Women reported lower (worse) mean (SD) WLI scores than men overall (52 [22] vs 57 [23]; mean difference, −5 [−0.2 SDs]; *P* < .001). In multivariable regression, lower WLI was independently associated with being a woman (linear regression coefficient, −6; SE, 0.7; *P* < .001) as well as being aged 35 years or older (eg, aged 35 to 44 years: linear regression coefficient, −7; SE, 1.4; *P* < .001), single (linear regression coefficient, −3 vs married; SE, 1.1; *P* = .003), working more hours (eg, 50 to 59 hours per week vs less than 40 hours per week: linear regression coefficient, −9; SE, 1.0; *P* < .001) and call nights (linear regression coefficient, −1 for each call night per week; SE, 0.2; *P* < .001), and being in emergency medicine (linear regression coefficient, −18; SE, 1.6, *P* < .001), urology (linear regression coefficient, −11; SE, 4.0; *P* = .009), general surgery (linear regression coefficient, −4; SE, 2.0; *P* = .04), anesthesiology (linear regression coefficient, −4; SE, 1.7; *P* = .03), or family medicine (linear regression coefficient, −3; SE, 1.4; *P* = .04) (reference category, internal medicine subspecialties). In interaction modeling, physician age, youngest child’s age, and hours worked per week modified the associations between gender and WLI, such that the largest gender disparities were observed in physicians who were aged 45 to 54 years (estimated WLI score for women, 49; 95% CI, 47-51; estimated WLI score for men, 57, 95% CI, 55-59; *P* < .001), had youngest child aged 23 years or older (estimated WLI score for women, 51; 95% CI, 48-54; estimated WLI score for men, 60; 95% CI, 58-62; *P* < .001), and were working less than 40 hours per week (estimated WLI score for women, 61; 95% CI, 59-63; estimated WLI score for men; 70; 95% CI, 68-72; *P* < .001).

**Conclusions and Relevance:**

This study found that lower WLI was reported by physicians who are women, single, aged 35 years or older, and who work more hours and call nights. These findings suggest that systemic change is needed to improve WLI among physicians.

## Key Points

**Question:**

Which personal and professional factors are independently associated with work-life integration in physicians, and which factors modify the association between gender and work-life integration?

**Findings:**

In this cross-sectional study based on survey data of 4370 US physicians, women physicians consistently reported significantly worse work-life integration scores independent of other personal and professional factors, with a gender disparity most pronounced for midcareer physicians, those with adult children, and those working fewer hours per week.

**Meaning:**

These findings suggest that systemic change is needed to help physicians achieve appropriate integration of work life and home responsibilities.

## Teaser

This cross-sectional study examines which personal and professional factors may be associated with lower work-life integration scores among US physicians.

## Introduction

The modern medical profession is characterized by long hours, inflexible schedules, emotionally taxing situations, and a culture of prioritizing patient care over personal needs that put health care workers at risk for conflicts between work and home responsibilities. These conflicts result in poor work-life integration (WLI), in which an individual must choose which of multiple competing interests to prioritize in light of limited time or other resources. Dissatisfaction with WLI is consistently more prevalent among physicians relative to the general working population and is strongly associated with burnout, intention to reduce work hours, and intention to leave practice.^1,2,3,4,5,6,7,8^ Physicians also more commonly report that their careers negatively impact relationships with their children relative to the general working population, an effect most pronounced for women physicians.^9^

Women physicians report more problems with WLI than men.^1,4,5,10,11^ At work, women physicians may spend more time with patients and more frequently address psychosocial issues.^12,13,14,15^ At home, women physicians also spend more time on both household and childcare activities and on completing work from home.^16,17,18^ This combination of increased burdens for women vs men physicians both at work and at home may explain much of the observed gender disparities in WLI.

However, the interaction of gender with other demographic and practice setting characteristics is poorly understood, particularly in association with poor WLI. Prior studies have examined overall physician satisfaction with WLI, but underlying specific WLI behaviors (eg, healthy eating or sleep habits) are not well described.^4,5,8,11,19^ Understanding and addressing WLI behaviors and their association with gender and other personal and professional characteristics may yield substantial benefits to both health care workers and their patients.^20,21,22^

This study sought to (1) identify the personal and professional factors independently associated with WLI and (2) identify factors that modify the association between gender and WLI in a large national sample of US physicians.

## Methods

We used a cross-sectional design to assess a wide range of personal and professional characteristics that may be associated with WLI. Measures relevant to this study were part of a larger survey on professional satisfaction among physicians. This cross-sectional study was approved by the institutional review boards at Mayo Clinic and Stanford University prior to data collection, and is presented in accordance with the Strengthening the Reporting of Observational Studies in Epidemiology (STROBE) reporting guideline.

### Study Participants

Survey administration procedures have previously been described in detail.^8^ In brief, we assembled a sample of US physicians from all specialties using the American Medical Association Physician Masterfile. To ensure adequate representation from physicians in all specialties, we oversampled physicians in fields other than family medicine, general internal medicine, general pediatrics, and obstetrics/gynecology in an effort to have an adequate number of physicians from each specialty. We sent an initial invitation email to 83 291 physicians in October 2017, followed by 4 reminder email requests over the following 6 weeks. We then followed this electronic survey with paper surveys sent to a random sample of 5000 nonresponders (1426 had opened an email invitation but did not complete the electronic survey, 80 of whom also had paper surveys returned as undeliverable; 3574 had not opened an e-mail, 189 of whom also had paper surveys returned as undeliverable). Finally, we sent paper surveys and a $20 incentive to participate to a random sample of 500 nonresponders, with a reminder paper survey 3 weeks later and an abbreviated paper survey 6 weeks later. The stated purpose of the study was to better understand the factors contributing to satisfaction among US physicians. Participation was voluntary, following written informed consent at the survey introduction, and all responses were anonymous. We considered the 30 456 physicians who opened an invitation email or received a paper mailing (ie, not returned as undeliverable) as having received an invitation to respond, and we included all surveys completed and returned by March 15, 2018, in the analysis.

### WLI Measures

We assessed WLI using a previously-published 8-item scale designed to assess individual differences in WLI behaviors.^22,23,24,25^ The 8 survey prompts each begin with the phrase “During the past week, how often did this occur?” They conclude with “...skipped a meal,” “…ate a poorly balanced meal,” “…worked through a shift with no breaks,” “…arrived home late from work,” “…had difficulty sleeping,” “…slept less than 5 hours in a night,” “…changed personal/family plans due to work,” and “…felt frustrated by technology.” The first 7 items focus on tangible frequencies of activities reflecting the interaction between work and personal responsibilities, whereas the 8th item serves as a key indicator of the ability of technology to facilitate efficient workflows and minimize work-home conflicts.^21^ Each item is scored on a 4-point Likert scale (“Rarely or none of the time,” “Some or a little of the time,” “Occasionally or a moderate amount of time,” and “All of the time”). This WLI scale has been used among large and diverse samples of health care workers, showing good internal consistency (Cronbach α is 0.83 in validation study, 0.81 in current data set),^21,23,25^ as well as improvements associated with interventions.^26,27,28^ For ease of analysis and interpretation, we transformed the reverse-coded mean score onto a 0 to 100 scale, with 0 indicating poor WLI (“All of the time” for all items) and 100 indicating favorable WLI (“Rarely or none of the time” for all items).

### Personal and Professional Characteristics

We assessed personal and professional characteristics by respondent self-report. Personal characteristics previously determined or hypothesized to be associated with WLI include age, gender, race (options defined by investigators), relationship status, parenting status, and age of youngest child. Professional characteristics include specialty, nights on call per week, hours worked per week, primary practice setting, and number of years in practice.

### Statistical Analysis

We used descriptive statistics including frequencies, means, and standard deviations to describe survey responses. We assessed for independent variables associated with the WLI score, starting with univariate linear regressions to screen for candidate variables using a cutoff value of *P* < .10. We then used multivariable linear regression with these screened variables to identify independent associations with WLI scores.

For interaction analysis we started with a univariate linear regression model using WLI as the dependent variable and gender as the independent variable, then selected any variables that changed the gender coefficient by at least 10% when added to this model. We then constructed a multivariable linear regression model with all these selected variables, excluding those with *P* > .10. We added interaction terms for each selected variable and gender, retaining those with *P* values < .05, then added other interaction terms hypothesized to be associated with WLI, retaining those with *P* < .05.

Reference values were set at the modal value (categorical variables) or the lowest value (ordinal variables). Statistical significance was set at 2-sided *P* < .05. Due to the hypothesis-generating nature of these analyses, we made no corrections for multiple testing. All statistical analyses were performed in Stata version 15.0 (StataCorp) from November 2019 to July 2020.

## Results

Of 30 456 physicians who received an invitation to respond, 5197 (17%) completed surveys, 4370 of which provided complete responses for use in the present analysis. Of the physicians who provided complete responses, 2719 were men, 3491 were White/Caucasian (80.8%), 3560 were married (82.4%), and the mean (SD) age was 52.3 (12.0) years. Personal and professional characteristics of the respondents are shown in Table 1 and have been described previously in detail.^8^ Previously-reported analyses found measured demographic characteristics of respondents to be similar to known demographics of all US physicians.^8^

**Table 1.** Respondent Characteristics and Work-Life Integration (WLI) Scores

Characteristic	Respondents, No. (%)	WLI, mean (SD)			*P* value (women vs men)
		All^a^	Women (n = 1637)	Men (n = 2719)	
Work-life integration score (0-100)	4370 (100)	55 (23)	52 (22)	57 (23)	<.001
Age, y					
<35	289 (6.7)	57 (21)	56 (20)	60 (22)	.09
35-44	1028 (23.8)	52 (22)	52 (21)	53 (23)	.30
45-54	998 (23.1)	52 (23)	51 (22)	53 (24)	.09
55-64	1240 (28.7)	55 (23)	52 (23)	56 (23)	.004
≥65	707 (16.4)	64 (22)	62 (21)	64 (22)	.37
Missing	56 (1.3)	53 (23)	41 (19)	60 (22)	.004
Racial background					
White/Caucasian	3491 (80.8)	55 (23)	53 (22)	57 (23)	<.001
Asian	541 (12.5)	55 (24)	54 (23)	57 (25)	.08
Black/African American	127 (2.9)	52 (22)	47 (22)	61 (19)	<.001
American Indian/Alaskan Native	20 (0.5)	57 (25)	66 (19)	51 (28)	.21
Pacific Islander/Native Hawaiian	23 (0.5)	58 (23)	58 (25)	58 (21)	.96
Hispanic or Latino	250 (5.8)	54 (23)	53 (22)	56 (24)	.32
Other	198 (4.6)	57 (23)	55 (22)	57 (23)	.59
Relationship status					
Married	3560 (82.4)	56 (23)	53 (22)	58 (23)	<.001
Single	500 (11.6)	50 (23)	49 (22)	51 (25)	.30
Partnered	187 (4.3)	53 (23)	53 (23)	53 (23)	.97
Widow/widower	50 (1.2)	58 (25)	53 (26)	68 (20)	.04
Missing	21 (0.5)	47 (20)	42 (16)	52 (24)	.34
Age of youngest child, y					
No children	779 (18.1)	52 (22)	52 (21)	53 (23)	.56
<5	649 (15.0)	54 (22)	53 (21)	55 (23)	.53
5-12	703 (16.1)	53 (23)	51 (22)	54 (23)	.07
13-18	577 (13.2)	53 (23)	52 (22)	54 (24)	.32
19-22	375 (8.6)	54 (23)	52 (23)	54 (23)	.44
≥23	1252 (28.6)	60 (23)	54 (24)	62 (22)	<.001
Years in practice					
≤18	2138 (49.1)	53 (22)	51 (22)	54 (23)	.005
>18	2218 (50.9)	58 (23)	55 (23)	59 (23)	<.001
Specialty					
Internal medicine subspecialty	504 (11.7)	55 (22)	53 (22)	56 (22)	.10
General internal medicine	349 (8.1)	55 (23)	52 (23)	57 (23)	.04
Psychiatry	343 (7.9)	61 (22)	57 (22)	65 (22)	<.001
Family medicine	334 (7.7)	54 (22)	51 (20)	57 (23)	.02
General surgery subspecialty	326 (7.5)	48 (23)	43 (22)	50 (23)	.02
Emergency medicine	241 (5.6)	46 (22)	39 (19)	49 (22)	<.001
Orthopedic surgery	226 (5.2)	55 (22)	48 (19)	56 (23)	.06
General pediatrics	222 (5.1)	62 (22)	58 (22)	68 (20)	.001
Anesthesiology	208 (4.8)	55 (23)	49 (25)	58 (21)	.008
Pediatric subspecialty	180 (4.2)	57 (21)	56 (20)	59 (21)	.30
Radiology	168 (3.9)	58 (23)	55 (25)	58 (23)	.35
Neurology	159 (3.7)	57 (24)	53 (21)	59 (26)	.16
Obstetrics and gynecology	153 (3.5)	52 (21)	52 (20)	51 (23)	.85
General surgery	132 (3.1)	48 (23)	46 (19)	48 (24)	.59
Ophthalmology	122 (2.8)	64 (23)	59 (24)	66 (22)	.11
Pathology	120 (2.8)	60 (21)	59 (20)	61 (23)	.52
Dermatology	111 (2.6)	62 (21)	60 (19)	65 (23)	.21
Physical medicine and rehabilitation	105 (2.4)	55 (24)	49 (23)	58 (24)	.049
Neurosurgery	52 (1.2)	48 (27)	46 (22)	48 (28)	.79
Radiation oncology	37 (0.9)	56 (22)	53 (28)	57 (20)	.66
Otolaryngology	36 (0.8)	54 (20)	47 (24)	56 (19)	.27
Urology	27 (0.6)	42 (21)	40 (20)	43 (22)	.79
Preventive medicine/occupational medicine	21 (0.5)	56 (22)	51 (23)	59 (22)	.44
Other	109 (2.5)	58 (24)	55 (22)	59 (25)	.47
Missing	33 (0.8)	56 (22)	40 (21)	63 (18)	.002
Primary practice setting					
Private practice	2099 (48.6)	57 (23)	54 (22)	58 (24)	<.001
Academic medical center	1184 (27.4)	53 (22)	52 (22)	54 (21)	.10
Veteran’s hospital	91 (2.1)	59 (24)	48 (23)	67 (21)	<.001
Active military practice	47 (1.1)	53 (26)	49 (25)	56 (26)	.34
Not in practice or retired	80 (1.8)	61 (24)	49 (25)	65 (22)	.005
Other	790 (18.3)	55 (23)	52 (22)	57 (24)	.002
Missing	27 (0.6)	42 (22)	35 (21)	45 (22)	.25
Hours worked per week					
<40	732 (16.9)	65 (22)	60 (22)	70 (22)	<.001
40-49	873 (20.2)	63 (21)	60 (20)	66 (21)	<.001
50-59	1070 (24.8)	57 (20)	52 (21)	59 (20)	<.001
60-69	930 (21.5)	49 (22)	45 (20)	51 (22)	<.001
70-79	330 (7.6)	43 (21)	44 (20)	42 (22)	.51
≥80	317 (7.3)	37 (22)	36 (21)	38 (22)	.41
Missing	66 (1.5)	49 (23)	42 (23)	53 (22)	.07
Nights on call per week					
0	1634 (37.8)	61 (22)	58 (22)	63 (22)	<.001
1	1059 (24.5)	55 (22)	51 (20)	57 (23)	<.001
2	609 (14.1)	52 (22)	48 (21)	54 (23)	.004
3	285 (6.6)	47 (23)	44 (21)	49 (23)	.12
4	148 (3.4)	48 (22)	44 (23)	50 (21)	.12
5	100 (2.3)	49 (24)	53 (23)	47 (24)	.27
6	13 (0.3)	44 (25)	19 (9)	49 (25)	.12
7	470 (10.9)	51 (24)	48 (24)	53 (24)	.02

^a^ Includes “women,” “men,” “other,” and missing gender.

The mean (SD) WLI score was 55 (23). Women reported lower (worse) scores than men (mean [SD] WLI score was 52 [22] for women vs 57 [23] for men; mean difference, −5 [−0.2 SDs]; *P* < .001). Responses to 6 of the 8 individual WLI items (“ate a poorly balanced meal,” “worked through a shift with no breaks,” “arrived home late from work,” “had difficulty sleeping,” “changed personal/family plans due to work,” and “felt frustrated by technology”) were lower among women than men. Responses to the other 2 items (“skipped a meal” and “slept less than 5 hours in a night”) did not differ by gender.

### Univariate Analysis

Unadjusted gender differences in WLI scores by personal characteristics are shown in Table 1. Women reported lower mean (SD) WLI than men in nearly all subgroups, with the largest differences observed among those who identify as Black/African American (47 [22] for women vs 61 [19] for men; mean difference, −14 [−0.7 SDs]; *P* < .001) and those with youngest child aged at least 19 years (53 [24] for women vs 60 [23] for men; mean difference, −8 [−0.3 SDs]; *P* < .001). Single parents of dependent children reported lower mean (SD) WLI scores than single physicians without children (46 [25] for single parents vs 52 [23] for single nonparents; mean difference, −6 [−0.3 SDs]; *P* = .049). This difference in mean (SD) WLI scores persisted for the subgroup of single mothers (44 [22] vs 51 [22] for other single women; mean difference, −7 [−0.3 SDs]; *P* = .02) but not of single fathers (50 [28] vs 52 [25] for other single men; mean difference, −2 [−0.1 SDs]; *P* = .54).

**Figure 1.**
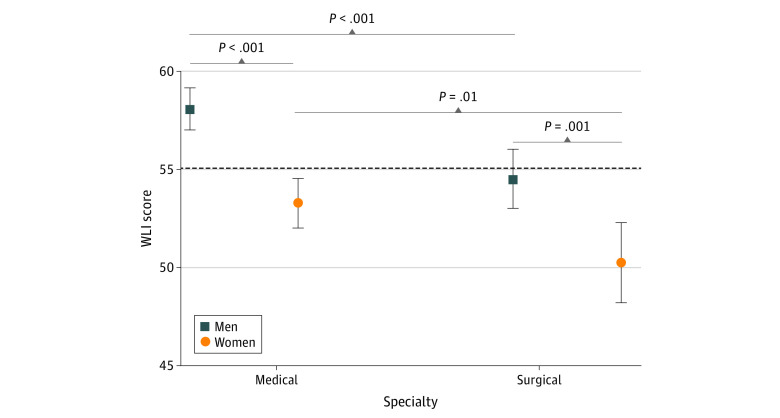
Work-Life Integration (WLI) Scores by Physician Sex and Specialty (Medical vs Surgical) Data shown as mean WLI scores with error bars denoting 95% CIs. *P*-values obtained via 2-tailed *t* tests. Reference line at the population mean of 55.

Unadjusted gender differences in WLI scores by professional characteristics are shown in Table 1 and eFigure 1, eFigure 2, eFigure 3, eFigure 4, and eFigure 5 in the Supplement. Women reported lower mean (SD) WLI than men in nearly all subgroups, with the largest differences observed among those who average fewer than 40 hours per week of work (60 [22] for women vs 70 [22] for men; mean difference, −10 [−0.5 SDs]; *P* < .001), those in emergency medicine (39 [19] for women vs 49 [22] for men; mean difference, −10 [−0.5 SDs]; *P* < .001), and those in general pediatrics (58 [22] for women vs 68 [20] for men; mean difference, −10 [−0.5 SDs]; *P* = .001). As shown in Figure 1, women reported lower mean (SD) WLI scores than men among pooled medical specialties (53 [22] vs 58 [23]; mean difference, −5 [−0.2 SDs]; *P* < .001) and pooled surgical specialties (50 [22] vs 54 [24]; mean difference, −4 [−0.2 SDs]; *P* = .001). Surgical respondents reported lower WLI than medical respondents, among men and women. Univariate associations with WLI are shown in eTable 1 in the Supplement.

### Multivariable Analysis

Results from the multivariable analysis are shown in Table 2. Women reported a 6-point lower WLI score than men (linear regression coefficient, −6; SE, 0.7; *P* < .001). Other independent variables associated with WLI were each of the 4 age categories older than the reference group of 34 years or younger (eg, aged 35 to 44 years: linear regression coefficient, −7; SE, 1.4; *P* < .001), single relationship status (linear regression coefficient, −3 vs married; SE, 1.1; *P* = .003) and years in practice (linear regression coefficient, +2 for each 10 years; SE, 1.0; *P* = .001). Compared with internal medicine subspecialties, lower WLI scores were reported in emergency medicine (linear regression coefficient, −18; SE, 1.6; *P* < .001), urology (linear regression coefficient, −11; SE, 4.0; *P* = .009), general surgery (linear regression coefficient, −4; SE, 2.0; *P* = .04), anesthesiology (linear regression coefficient, −4; SE, 1.7; *P* = .03), and family medicine (linear regression coefficient, −3; SE, 1.4; *P* = .04). Working more hours per week (eg, 50 to 59 hours per week vs less than 40 hours per week: linear regression coefficient, −9; SE, 1.0; *P* < .001) and working more frequent call nights per week (linear regression coefficient, −1 for each call night per week; SE, 0.2; *P* < .001) were also independently associated with lower WLI. Practice setting and age of youngest child were not associated with WLI in multivariable analysis.

**Table 2.** Multivariable Linear Regression Showing Personal and Professional Factors as Independent Variables Associated With Work-Life Integration^a^

Variable	Coefficient (SE)	*P* value	Overall *P* value^b^
Intercept	75 (2.0)	<.001	
Gender (vs man)	0	NA	
Woman	−6 (0.7)	<.001	<.001
Other	−20 (6.8)	.003	
Age (vs <35), y	0	NA	
35-44	−7 (1.4)	<.001	<.001
45-54	−8 (1.7)	<.001	
55-64	−8 (2.0)	<.001	
≥65	−7 (2.5)	.008	
Relationship status (vs married)	0	NA	
Single	−3 (1.1)	.003	.02
Partnered	−3 (1.6)	.09	
Widow/widower	0 (3.0)	.91	
Youngest child’s age (vs no children), y	0	NA	
<5	0 (1.2)	.91	.81
5-12	−1 (1.2)	.44	
13-18	−1 (1.3)	.42	
19-22	−1 (1.5)	.58	
≥23	0 (1.3)	.74	
Years in practice (per 10 y)	2 (1.0)	.001	
Specialty (vs internal medicine subspecialty)	0	NA	
Emergency medicine	−18 (1.6)	<.001	<.001
Urology	−11 (4.0)	.009	
General surgery	−4 (2.0)	.04	
Preventive medicine/occupational medicine	−4 (4.4)	.35	
Anesthesiology	−4 (1.7)	.03	
Family medicine	−3 (1.4)	.04	
Physical medicine and rehabilitation	−3 (2.2)	.18	
General surgery subspecialty	−3 (1.4)	.06	
Otolaryngology	−2 (3.5)	.56	
Obstetrics and gynecology	−2 (1.9)	.40	
Orthopedic surgery	−1 (1.6)	.45	
Radiation oncology	−1 (3.5)	.80	
Radiology	−1 (1.8)	.64	
Neurosurgery	0 (3.0)	.91	
General internal medicine	0 (1.4)	.84	
Neurology	0 (1.8)	.94	
Psychiatry	1 (1.4)	.45	
General pediatrics	2 (1.7)	.34	
Pediatric subspecialty	2 (1.8)	.34	
Dermatology	2 (2.1)	.29	
Pathology	3 (2.1)	.15	
Ophthalmology	3 (2.0)	.13	
Other	−2 (2.1)	.42	
Missing	1 (3.6)	.77	
Practice setting (vs private practice)	0	NA	
Academic medical center	0 (0.8)	.60	.047
Veteran’s hospital	−1 (2.2)	.74	
Active military practice	1 (3.0)	.78	
Hours worked per week (vs <40 h)	0	NA	
40-49	−2 (1.0)	.09	<.001
50-59	−9 (1.0)	<.001	
60-69	−16 (1.1)	<.001	
70-79	−22 (1.4)	<.001	
≥80	−27 (1.5)	<.001	
Call nights per week (per night)	−1 (0.2)	<.001	

Abbreviation: NA, not applicable.

^a^ N = 4370 respondents. Dependent variable is work-life integration score (0-100 point scale). Estimates via multivariable linear regression with all covariates shown.

^b^ Overall *P*-values for categorical variables via Wald test.

### Interaction Analysis

**Figure 2.**
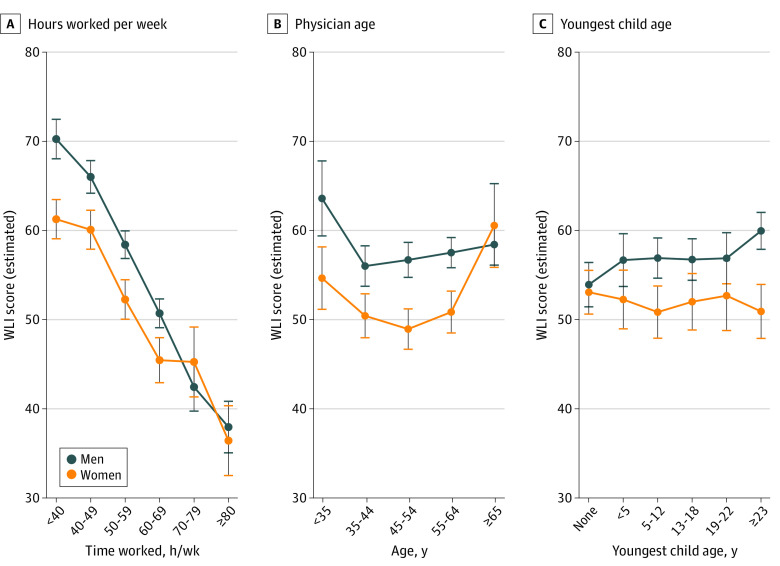
Multivariable Interaction Models Estimating Work-Life Integration (WLI) Scores Estimated WLI scores showing the interactions between gender and (A) mean hours worked per week, (B) physician age in years, and (C) age of youngest child in years. Models also adjusted for relationship status and specialty. Error bars denote 95% CIs.

In interaction modeling, the association between gender and WLI was modified by physician age, youngest child’s age, and hours worked per week, after adjustment for specialty, relationship status, and the interaction between relationship status and youngest child’s age (eTable 2 in Supplement). As shown in Figure 2, the gender disparity in WLI was smaller with increasing hours worked per week, with no differences observed beyond 70 hours worked per week. The gender disparity was present in all physician age categories except for those aged 65 years or older, but was U-shaped with the most pronounced disparity in the age category of 45 to 54 years. Quantitatively, the largest gender disparities were observed in physicians who were aged 45 to 54 years (estimated WLI score for women, 49; 95% CI, 47-51; estimated WLI score for men, 57, 95% CI, 55-59; *P* < .001), had youngest child aged 23 years or older (estimated WLI score for women, 51; 95% CI, 48-54; estimated WLI score for men, 60; 95% CI, 58-62; *P* < .001), and were working less than 40 hours per week (estimated WLI score for women, 61; 95% CI 59-63; estimated WLI score for men; 70; 95% CI 68-72; *P* < .001). These findings are qualitatively similar to the unadjusted findings, shown in eFigure 5, eFigure 6, and eFigure 7 in the Supplement.

## Discussion

This cross-sectional study found that lower (worse) WLI is most frequently reported by physicians who are women, aged 35 years or older, single, and those who work longer hours and more call nights per week. Emergency medicine and urology physicians exhibited the lowest WLI scores overall, whereas emergency medicine physicians and general pediatricians exhibited the largest disparity between women’s and men’s WLI scores. Hours worked per week, physician age, and age of the youngest child also modified the association between gender and WLI.

A pattern of lower WLI among women was common across personal and professional categories and persisted in multivariable analyses. “Home-at-work” (eg, a new mother pumping breastmilk while at work) and “work-at-home” (eg, finishing clinical documentation at home in the evening) activities are common and have been associated with adverse effects on physician well-being, underscoring the importance of understanding drivers of WLI and its disparities among physicians.^2,3,29,30^ Physician mothers report nearly 2 more hours spent on household activities per day than physician fathers, primarily due to additional cleaning, food preparation, and childcare time.^18,31,32^ Childcare responsibilities do not occur exclusively outside of work hours, and attending doctor or dentist visits, participating in school functions, or transporting to after-school activities may require reallocation of work time from the daytime into the evenings or late night.^33^ For physicians in positions without flexibility for time reallocation, these responsibilities may be additionally taxing. The gender disparity in WLI was most pronounced among parents of adult children less likely to require direct assistance with daily activities, which may be reflective of generational norms or indicate other disproportionate time demands unrelated to parenting. The gender disparity was also most pronounced among those working the fewest hours per week, indicating that even women physicians working relatively few hours are more likely than men to make sacrifices reflected in the WLI scale.

Compared with married respondents, a relationship status of “single” was associated with lower WLI. Single physicians, especially those who live alone, have less opportunity to share household responsibilities with others, which may be particularly challenging for single parent physicians when combined with long or irregular work hours.^34^ Although relationship status did not significantly modify the gender disparity in WLI, it did interact with youngest child’s age such that single parents of young children had lower WLI. Furthermore, single physicians may also have increased demands for their time for many other reasons, including if they cover clinical responsibilities for colleagues who perceive that they have more flexible schedules, if they are in a nonpartnered relationship (which may require long-distance travel), or if they are seeking a relationship.^34^

The age categories of 35 to 44 years and 45 to 54 years had the worst WLI scores both for men and women, consistent with prior findings of the lowest satisfaction with WLI among midcareer physicians.^35^ This trend may reflect a tendency toward increased work hours or less favorable schedules, increased home responsibilities among those with dependent children, frustrations with adapting to changing practice environments, or expansion of administrative duties among midcareer physicians.^35^ In addition, a general tendency toward improving self-management of WLI over time, or selective attrition of physicians with poor WLI may explain the relatively better WLI scores among late-career physicians.^36,37^ Not surprisingly, increased hours worked and call nights per week were associated with lower WLI, as these reflect direct reductions in time available for home responsibilities.

Poor WLI may have adverse effects on physicians and their families at multiple levels, highlighting the urgency of addressing these disparities. Women are more likely to experience burnout, which may be largely attributable to differences in professional characteristics and satisfaction with WLI.^1,3,11^ Women report more general dissatisfaction with WLI relative to men, both in private practice and academic practice, when assessed using a single-item measure of agreement with “My work schedule leaves me enough time for my personal/family life.”^11^ This measure may be influenced both by an individual’s expectations of their work schedule and by their thresholds for satisfaction, reflecting that WLI is a phenomenon in which work culture intersects with personal values, and underscoring the notion that societal and organizational change will be needed to drive lasting progress toward equality.

Although societal norms may be slow to shift, our findings suggest several potential mechanisms that organizations may use in an attempt to accelerate change and reduce gender disparities among those who are most affected. Increased control in scheduling, both in the distribution and volume of patient care hours, may reduce the frequency of work-home conflict.^1,38,39^ Although overall patient care demands on the physician workforce are unlikely to recede based on predicted physician shortages, practice efficiency improvements may maximize limited physician time while continuing to meet the societal need for health care (ie, via redistribution of practice structures or prioritization of team-based care).^1,40,41,42,43,44,45,46,47^ Any such interventions likely will be most effective if they are designed to also decrease the well-documented gender disparities related to compensation, retention, and promotion, as these disparities can encourage women to take on excess work and to perceive their work as less valuable than their male colleagues.^2,48,49,50,51^ Gender-specific mentorship, coaching, and networking may assist women physicians in recognizing and addressing the unique pressures placed on them.^17,52,53,54^ On-site or other readily accessible high quality backup childcare may also reduce gender disparities among physician-parents, particularly for holidays or for prolonged school closures such as experienced during the COVID-19 pandemic in 2020.^1,2,17,38^

### Limitations

This study had some limitations and must be interpreted in light of its design. Although the WLI scale targets a more comprehensive and less subjective assessment than the single item measure of satisfaction with WLI, the interpretation of individual items remains up to the respondent and may be somewhat affected by an individual’s interpretation of societal expectations, whether or not those expectations exist (eg, “changing personal/family plans because of work” may take on differential meanings for men vs women or for military vs civilian physicians).^55^ There is no identified threshold for distinguishing “acceptable” from “unacceptable” WLI, but empirically considering 0.5 standard deviations meaningful would approximate to an 11-point difference among this study sample. As a cross-sectional observational study, we cannot determine causality of the observed relationships, or the potential direction of any such effect. Despite extensive evaluation that found no clear evidence of response bias (published separately),^8^ it remains possible that physicians with poor WLI may have been more or less likely to respond to the survey. Survey items were self-reported, measures such as work hours and call nights may be subject to recall bias, and there may be other important factors related to WLI not measured here, including geographic distribution and distribution of clinical vs nonclinical professional roles. Although the 14 individuals who identified their gender as other indicated worse WLI than men or women, this cohort was too small to make meaningful conclusions regarding the experiences of individuals who identify as other. Further research is needed to better understand the experiences of this diverse group of physicians.

## Conclusions

In conclusion, WLI is an important aspect of physician well-being, but physicians have differing experiences of WLI by gender, age, relationship status, specialty, and work hours. Women physicians consistently report lower WLI across a range of ages, children’s ages, and work hours. This study’s findings suggest that systemic change is needed to enable physicians to achieve appropriate integration of work life and home responsibilities.

## Notes

Supplement. **eFigure 1.** Work-Life Integration for Surgical Specialties, for Males (X-axis) and Females (Y-axis) **eFigure 2.** Work-Life Integration for General Medical Specialties, for Males (X-axis) and Females (Y-axis) **eFigure 3.** Work-Life Integration for Medical Subspecialties, for Males (X-axis) and Females (Y-axis) **eFigure 4.** Work-Life Integration by Practice Setting, for Males (X-axis) and Females (Y-axis) **eFigure 5.** Work-Life Integration by Hours Worked Per Week, for Males (X-axis) and Females (Y-axis) **eFigure 6.** Work-Life Integration by Physician Sex and Physician Age **eFigure 7.** Work-Life Integration by Physician Sex and Youngest Child’s Age in Years **eTable 1.** Univariate Screen of Variables Associated With Work-Life Integration **eTable 2.** Interaction Model. Multivariable Linear Regression Showing Personal/Professional Factors and Interactions as Independent Variables Associated With Work-Life Integration

## References

[1] Dyrbye LN, Shanafelt TD, Balch CM, Satele D, Sloan J, Freischlag J. Relationship between work-home conflicts and burnout among American surgeons: a comparison by sex. Arch Surg. 2011;146(2):211-217. doi:10.1001/archsurg.2010.310 21339435

[2] Dyrbye LN, Sotile W, Boone S, . A survey of U.S. physicians and their partners regarding the impact of work-home conflict. J Gen Intern Med. 2014;29(1):155-161. doi:10.1007/s11606-013-2581-3 24043567 PMC3889954

[3] Dyrbye LN, West CP, Satele D, Sloan JA, Shanafelt TD. Work/Home conflict and burnout among academic internal medicine physicians. Arch Intern Med. 2011;171(13):1207-1209. doi:10.1001/archinternmed.2011.289 21747018

[4] Shanafelt TD, Boone S, Tan L, . Burnout and satisfaction with work-life balance among US physicians relative to the general US population. Arch Intern Med. 2012;172(18):1377-1385. doi:10.1001/archinternmed.2012.3199 22911330

[5] Shanafelt TD, Hasan O, Dyrbye LN, . Changes in burnout and satisfaction with work-life balance in physicians and the general US working population between 2011 and 2014. Mayo Clin Proc. 2015;90(12):1600-1613. doi:10.1016/j.mayocp.2015.08.023 26653297

[6] Sinsky CA, Dyrbye LN, West CP, Satele D, Tutty M, Shanafelt TD. Professional satisfaction and the career plans of US physicians. Mayo Clin Proc. 2017;92(11):1625-1635. doi:10.1016/j.mayocp.2017.08.017 29101932

[7] Dyrbye LN, Freischlag J, Kaups KL, . Work-home conflicts have a substantial impact on career decisions that affect the adequacy of the surgical workforce. Arch Surg. 2012;147(10):933-939. doi:10.1001/archsurg.2012.835 23117833

[8] Shanafelt TD, West CP, Sinsky C, . Changes in burnout and satisfaction with work-life integration in physicians and the general US working population between 2011 and 2017. Mayo Clin Proc. 2019;94(9):1681-1694. doi:10.1016/j.mayocp.2018.10.023 30803733

[9] Shanafelt TD, Hasan O, Hayes S, . Parental satisfaction of U.S. physicians: associated factors and comparison with the general U.S. working population. BMC Med Educ. 2016;16(1):228. doi:10.1186/s12909-016-0737-7 27567665 PMC5002113

[10] Guille C, Frank E, Zhao Z, . Work-family conflict and the sex difference in depression among training physicians. JAMA Intern Med. 2017;177(12):1766-1772. doi:10.1001/jamainternmed.2017.5138 29084311 PMC5820732

[11] Marshall AL, Dyrbye LN, Shanafelt TD, . Disparities in burnout and satisfaction with work-life integration in U.S. Physicians by gender and practice setting. Acad Med. 2020;95(9):1435-1443. doi:10.1097/ACM.0000000000003521 32459677

[12] Dahlke AR, Johnson JK, Greenberg CC, . Gender Differences in utilization of duty-hour regulations, aspects of burnout, and psychological well-being among general surgery residents in the United States. Ann Surg. 2018;268(2):204-211. doi:10.1097/SLA.0000000000002700 29462009

[13] Roter DL, Hall JA, Aoki Y. Physician gender effects in medical communication: a meta-analytic review. JAMA. 2002;288(6):756-764. doi:10.1001/jama.288.6.756 12169083

[14] Wu D, Gross B, Rittenhouse K, Harnish C, Mooney C, Rogers FB. A preliminary analysis of compassion fatigue in a surgeon population: are female surgeons at heightened risk? Am Surg. 2017;83(11):1302-1307. doi:10.1177/000313481708301136 29183536

[15] Zhang LM, Ellis RJ, Ma M, . Prevalence, types, and sources of bullying reported by US general surgery residents in 2019. JAMA. 2020;323(20):2093-2095. doi:10.1001/jama.2020.2901 32453357 PMC7251443

[16] Starmer AJ, Frintner MP, Matos K, Somberg C, Freed G, Byrne BJ. Gender discrepancies related to pediatrician work-life balance and household responsibilities. Pediatrics. 2019;144(4):e20182926. doi:10.1542/peds.2018-2926 31506304

[17] Templeton KC, Bernstein J, Sukhera LM, Gender-based differences in burnout: issues faced by women physicians. National Academy of Medicine. Published May 30, 2019. Accessed April 8, 2021. https://nam.edu/gender-based-differences-in-burnout-issues-faced-by-women-physicians/

[18] Jolly S, Griffith KA, DeCastro R, Stewart A, Ubel P, Jagsi R. Gender differences in time spent on parenting and domestic responsibilities by high-achieving young physician-researchers. Ann Intern Med. 2014;160(5):344-353. doi:10.7326/M13-0974 24737273 PMC4131769

[19] Garcia LC, Shanafelt TD, West CP, . Burnout, depression, career satisfaction, and work-life integration by physician race/ethnicity. JAMA Netw Open. 2020;3(8):e2012762. doi:10.1001/jamanetworkopen.2020.12762 32766802 PMC7414389

[20] Hämmig O, Brauchli R, Bauer GF. Effort-reward and work-life imbalance, general stress and burnout among employees of a large public hospital in Switzerland. Swiss Med Wkly. 2012;142:w13577. doi:10.4414/smw.2012.13577 22653680

[21] Schwartz SP, Adair KC, Bae J, . Work-life balance behaviours cluster in work settings and relate to burnout and safety culture: a cross-sectional survey analysis. BMJ Qual Saf. 2019;28(2):142-150. doi:10.1136/bmjqs-2018-007933 30309912 PMC6365921

[22] Sexton JB, Schwartz SP, Chadwick WA, . The associations between work-life balance behaviours, teamwork climate and safety climate: cross-sectional survey introducing the work-life climate scale, psychometric properties, benchmarking data and future directions. BMJ Qual Saf. 2017;26(8):632-640. doi:10.1136/bmjqs-2016-006032 28008006 PMC5481495

[23] Sexton JB, Adair KC, Leonard MW, . Providing feedback following Leadership WalkRounds is associated with better patient safety culture, higher employee engagement and lower burnout. BMJ Qual Saf. 2018;27(4):261-270. doi:10.1136/bmjqs-2016-006399 28993441 PMC5867443

[24] Adair KC, Quow K, Frankel A, . The Improvement Readiness scale of the SCORE survey: a metric to assess capacity for quality improvement in healthcare. BMC Health Serv Res. 2018;18(1):975. doi:10.1186/s12913-018-3743-0 30558593 PMC6296100

[25] Sexton JB. Technical Report 16-8: SCORE: Assessment of your work setting Safety, Communication, Operational Reliability, and Engagement. 2017. Accessed December 18, 2019. https://www.hsq.dukehealth.org/files/2019/05/SCORE_Technical_Report_5.14.19.pdf

[26] Sexton JB, Adair KC. Forty-five good things: a prospective pilot study of the Three Good Things well-being intervention in the USA for healthcare worker emotional exhaustion, depression, work-life balance and happiness. BMJ Open. 2019;9(3):e022695. doi:10.1136/bmjopen-2018-022695 30898795 PMC6475256

[27] Adair KC, Kennedy LA, Sexton JB. Three good tools: positively reflecting backwards and forwards is associated with robust improvements in well-being across three distinct interventions. J Posit Psychol. 2020;15(5):613-622. doi:10.1080/17439760.2020.1789707 PMC8294345 34295357

[28] Adair KC, Rodriguez-Homs LG, Masoud S, Mosca PJ, Sexton JB. Gratitude at work: prospective cohort study of a web-based, single-exposure well-being intervention for health care workers. J Med Internet Res. 2020;22(5):e15562. doi:10.2196/15562 32406864 PMC7256751

[29] Grinberg C. Pumped. JAMA. 2018;320(10):977-978. doi:10.1001/jama.2018.12212 30208459

[30] Linzer M, Poplau S, Babbott S, . Worklife and wellness in academic general internal medicine: results from a national survey. J Gen Intern Med. 2016;31(9):1004-1010. doi:10.1007/s11606-016-3720-4 27138425 PMC4978678

[31] Ly DP, Jena AB. Sex differences in time spent on household activities and care of children among US physicians, 2003-2016. Mayo Clin Proc. 2018;93(10):1484-1487. doi:10.1016/j.mayocp.2018.02.018 29673711 PMC8099448

[32] Baptiste D, Fecher AM, Dolejs SC, . Gender differences in academic surgery, work-life balance, and satisfaction. J Surg Res. 2017;218:99-107. doi:10.1016/j.jss.2017.05.075 28985884

[33] Raffi J, Trivedi MK, White L, Murase JE. Work-life balance among female dermatologists. Int J Womens Dermatol. 2019;6(1):13-19. doi:10.1016/j.ijwd.2019.07.001 32025555 PMC6997827

[34] Antonoff MB, Brown LM. Work-life balance: the female cardiothoracic surgeon’s perspective. J Thorac Cardiovasc Surg. 2015;150(6):1416-1421. doi:10.1016/j.jtcvs.2015.09.057 26478231

[35] Dyrbye LN, Varkey P, Boone SL, Satele DV, Sloan JA, Shanafelt TD. Physician satisfaction and burnout at different career stages. Mayo Clin Proc. 2013;88(12):1358-1367. doi:10.1016/j.mayocp.2013.07.016 24290109

[36] Huber TS. Professionalism and the work-life balance. J Vasc Surg. 2014;60(4):1072-1082. doi:10.1016/j.jvs.2014.04.077 25135876

[37] Leigh JP, Kravitz RL, Schembri M, Samuels SJ, Mobley S. Physician career satisfaction across specialties. Arch Intern Med. 2002;162(14):1577-1584. doi:10.1001/archinte.162.14.1577 12123400

[38] Clemen NM, Blacker BC, Floen MJ, Schweinle WE, Huber JN. Work-life balance in women physicians in South Dakota: results of a state-wide assessment survey. S D Med. 2018;71(12):550-558. 30835988

[39] Cheesborough JE, Gray SS, Bajaj AK. Striking a better integration of work and life: challenges and solutions. Plast Reconstr Surg. 2017;139(2):495-500. doi:10.1097/PRS.0000000000002955 28125538

[40] Association of American Medical Colleges. The complexities of physician supply and demand: projections from 2018 to 2033. Published June 2020. Accessed April 8, 2021. https://www.aamc.org/media/45976/download

[41] Zhang X, Lin D, Pforsich H, Lin VW. Physician workforce in the United States of America: forecasting nationwide shortages. Hum Resour Health. 2020;18(1):8. doi:10.1186/s12960-020-0448-3 32029001 PMC7006215

[42] US Department of Health and Human Services, Health Resources and Services Administration, National Center for Health Workforce Analysis. State-level projections of supply and demand for primary care practitioners: 2013-2025. Published 2016. Accessed April 8, 2021. https://bhw.hrsa.gov/sites/default/files/bureau-health-workforce/data-research/primary-care-state-projections2013-2025.pdf

[43] Dastagir MT, Chin HL, McNamara M, Poteraj K, Battaglini S, Alstot L. Advanced proficiency EHR training: effect on physicians’ EHR efficiency, EHR satisfaction and job satisfaction. AMIA Annu Symp Proc. 2012;2012:136-143. 23304282 PMC3540432

[44] Shanafelt T, Goh J, Sinsky C. The business case for investing in physician well-being. JAMA Intern Med. 2017;177(12):1826-1832. doi:10.1001/jamainternmed.2017.4340 28973070

[45] Tawfik DS, Profit J, Webber S, Shanafelt TD. Organizational factors affecting physician well-being. Curr Treat Options Pediatr. 2019;5(1):11-25. doi:10.1007/s40746-019-00147-6 31632895 PMC6801108

[46] Shanafelt TD, Noseworthy JH. Executive leadership and physician well-being: nine organizational strategies to promote engagement and reduce burnout. Mayo Clin Proc. 2017;92(1):129-146. doi:10.1016/j.mayocp.2016.10.004 27871627

[47] National Academy of Medicine, National Academies of Sciences, Engineering, and Medicine. Taking Action Against Clinician Burnout: A Systems Approach to Professional Well-Being. The National Academies Press;2019. 31940160

[48] Kelly EL, Moen P, Tranby E. Changing workplaces to reduce work-family conflict: schedule control in a white-collar organization. Am Sociol Rev. 2011;76(2):265-290. doi:10.1177/0003122411400056 21580799 PMC3094103

[49] Horowitz E, Feldman HA, Savich R. Neonatologist salary: factors, equity and gender. J Perinatol. 2019;39(3):359-365. doi:10.1038/s41372-018-0304-7 30617285

[50] Jena AB, Olenski AR, Blumenthal DM. Sex differences in physician salary in US public medical schools. JAMA Intern Med. 2016;176(9):1294-1304. doi:10.1001/jamainternmed.2016.3284 27400435 PMC5558151

[51] Byington CL, Lee V. Addressing disparities in academic medicine: moving forward. JAMA. 2015;314(11):1139-1141. doi:10.1001/jama.2015.10664 26372582

[52] Strong EA, De Castro R, Sambuco D, . Work-life balance in academic medicine: narratives of physician-researchers and their mentors. J Gen Intern Med. 2013;28(12):1596-1603. doi:10.1007/s11606-013-2521-2 23765289 PMC3832709

[53] Girod S, Fassiotto M, Grewal D, . Reducing implicit gender leadership bias in academic medicine with an educational intervention. Acad Med. 2016;91(8):1143-1150. doi:10.1097/ACM.0000000000001099 26826068

[54] Schueller-Weidekamm C, Kautzky-Willer A. Challenges of work-life balance for women physicians/mothers working in leadership positions. Gend Med. 2012;9(4):244-250. doi:10.1016/j.genm.2012.04.002 22626768

[55] Lin KY, Burgard SA. Working, parenting and work-home spillover: gender differences in the work-home interface across the life course. Adv Life Course Res. 2018;35:24-36. doi:10.1016/j.alcr.2017.12.003 29910698 PMC5997267

