# Supplementary material for: Personal and Professional Factors Associated With Work-Life Integration Among US Physicians
Source: JAMA Netw Open. 2021 May 27;4(5):e2111575. doi: 10.1001/jamanetworkopen.2021.11575 (PMC8160595; doi:10.1001/jamanetworkopen.2021.11575)
Supplement: Supplement. — eFigure 1. Work-Life Integration for Surgical Specialties, for Males (X-axis) and Females (Y-axis) eFigure 2. Work-Life Integration for General Medical Specialties, for Males (X-axis) and Females (Y-axis) eFigure 3. Work-Life Integration for Medical Subspecialties, for Males (X-axis) and Females (Y-axis) eFigure 4. Work-Life Integration by Practice Setting, for Males (X-axis) and Females (Y-axis) eFigure 5. Work-Life Integration by Hours Worked Per Week, for Males (X-axis) and Females (Y-axis) eFigure 6. Work-Life Integration by Physician Sex and Physician Age eFigure 7. Work-Life Integration by Physician Sex and Youngest Child’s Age in Years eTable 1. Univariate Screen of Variables Associated With Work-Life Integration eTable 2. Interaction Model. Multivariable Linear Regression Showing Personal/Professional Factors and Interactions as Independent Variables Associated With Work-Life Integration [file jamanetwopen-e2111575-s001.pdf]

Supplemental Online Content

Tawfik DS, Shanafelt TD, Dyrbye LN, et al. Personal and professional factors associated with work-life integration among US physicians. *JAMA Netw Open*. 2021;4(5):e2111575. doi:10.1001/jamanetworkopen.2021.11575

eFigure 1. Work-Life Integration for Surgical Specialties, for Males (X-axis) and Females (Y-axis)

eFigure 2. Work-Life Integration for General Medical Specialties, for Males (X-axis) and Females (Y-axis)

eFigure 3. Work-Life Integration for Medical Subspecialties, for Males (X-axis) and Females (Y-axis)

eFigure 4. Work-Life Integration by Practice Setting, for Males (X-axis) and Females (Y-axis)

eFigure 5. Work-Life Integration by Hours Worked Per Week, for Males (X-axis) and Females (Y-axis)

eFigure 6. Work-Life Integration by Physician Sex and Physician Age

eFigure 7. Work-Life Integration by Physician Sex and Youngest Child's Age in Years

eTable 1. Univariate Screen of Variables Associated With Work-Life Integration

eTable 2. Interaction Model. Multivariable Linear Regression Showing Personal/Professional Factors and Interactions as Independent Variables Associated With Work-Life Integration

This supplemental material has been provided by the authors to give readers additional information about their work.

© 2021 Tawfik DS et al. *JAMA Network Open.*

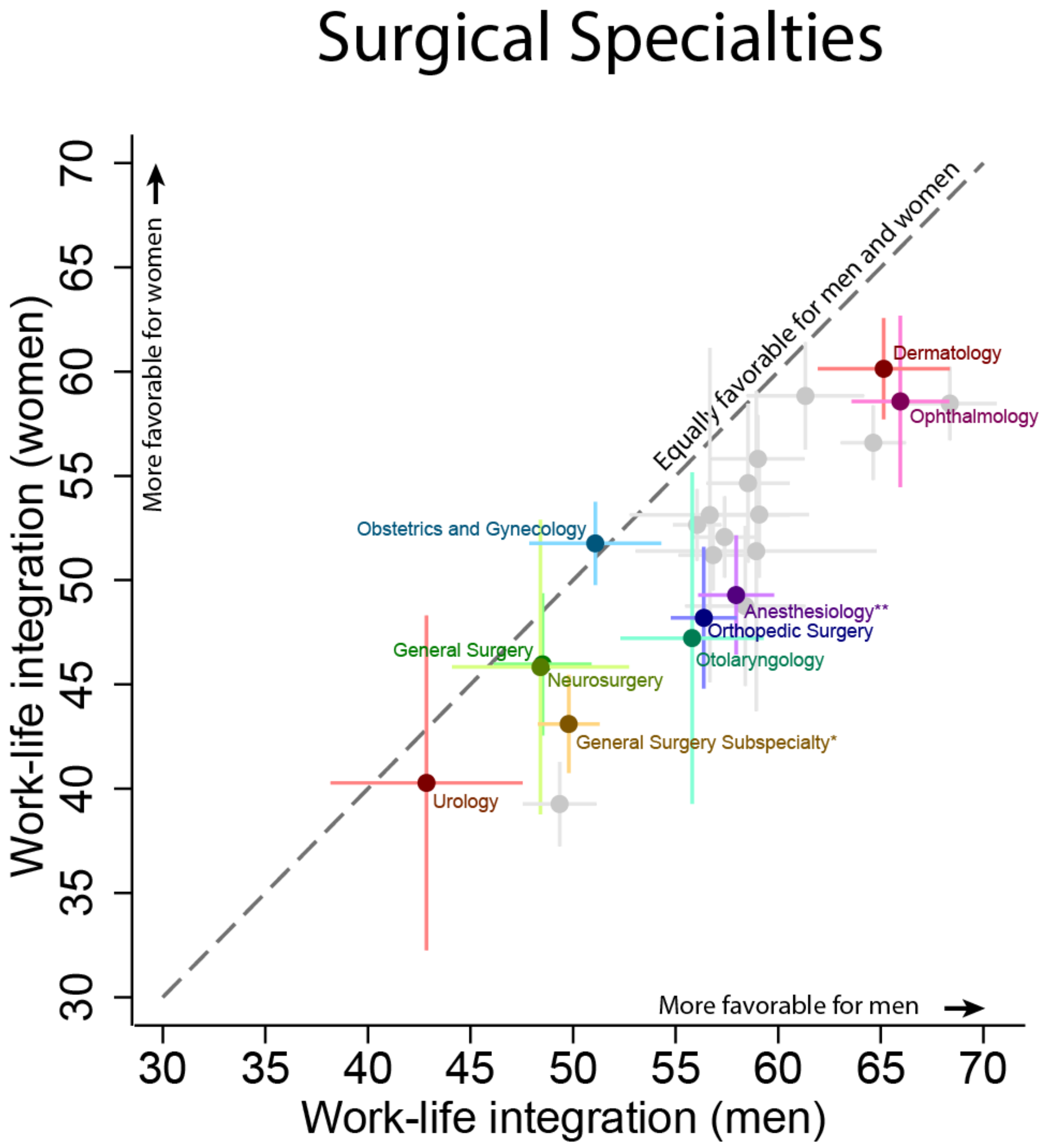

eFigure 1. Work-life integration for surgical specialties, for males (x-axis) and females (y-axis), shown as means and standard errors. Asterisks denote significantly different work-life integration scores between male and female respondents, via two-tailed t-test. * indicates $P < .05$; ** indicates $P < .01$; *** indicates $P < .001$

© 2021 Tawfik DS et al. *JAMA Network Open.*

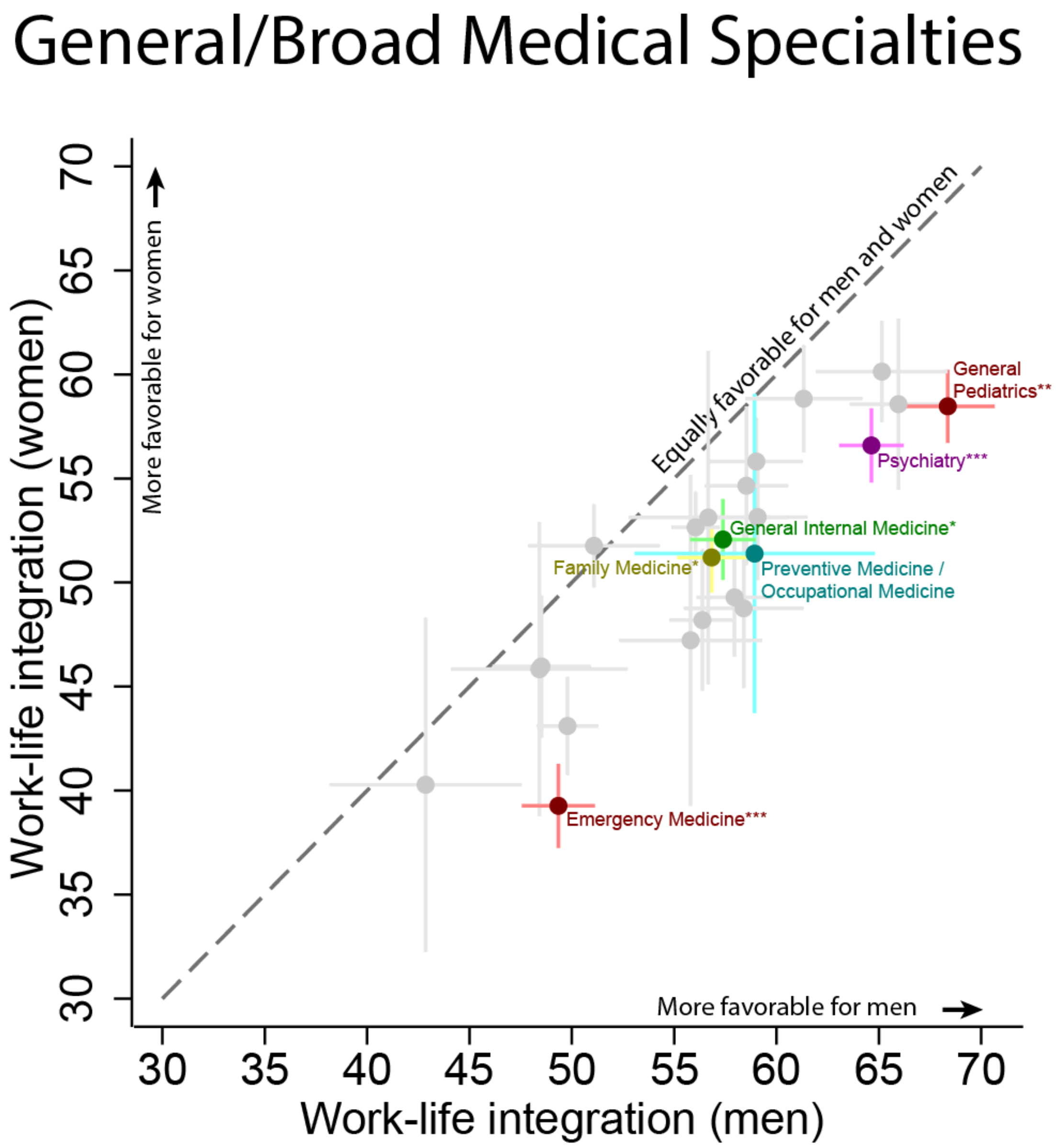

eFigure 2. Work-life integration for general medical specialties, for males (x-axis) and females (y-axis), shown as means and standard errors. Asterisks denote significantly different work-life integration scores between male and female respondents, via two-tailed t-test. * indicates *P* < .05; ** indicates *P* < .01; *** indicates *P* < .001

© 2021 Tawfik DS et al. *JAMA Network Open.*

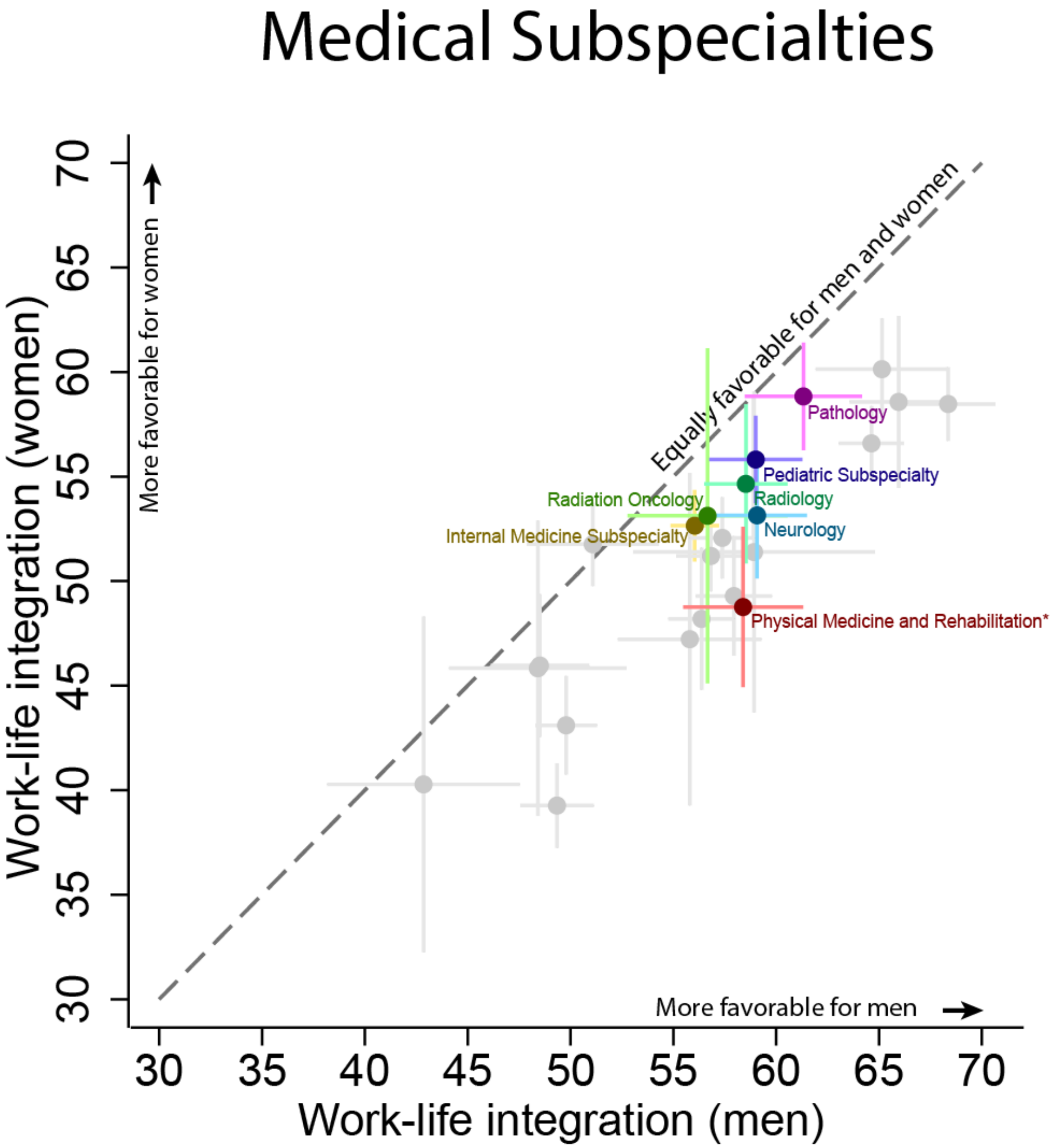

eFigure 3. Work-life integration for medical subspecialties, for males (x-axis) and females (y-axis), shown as means and standard errors. Asterisks denote significantly different work-life integration scores between male and female respondents, via two-tailed t-test. * indicates *P* < .05; ** indicates *P* < .01; *** indicates *P* < .001

© 2021 Tawfik DS et al. *JAMA Network Open.*

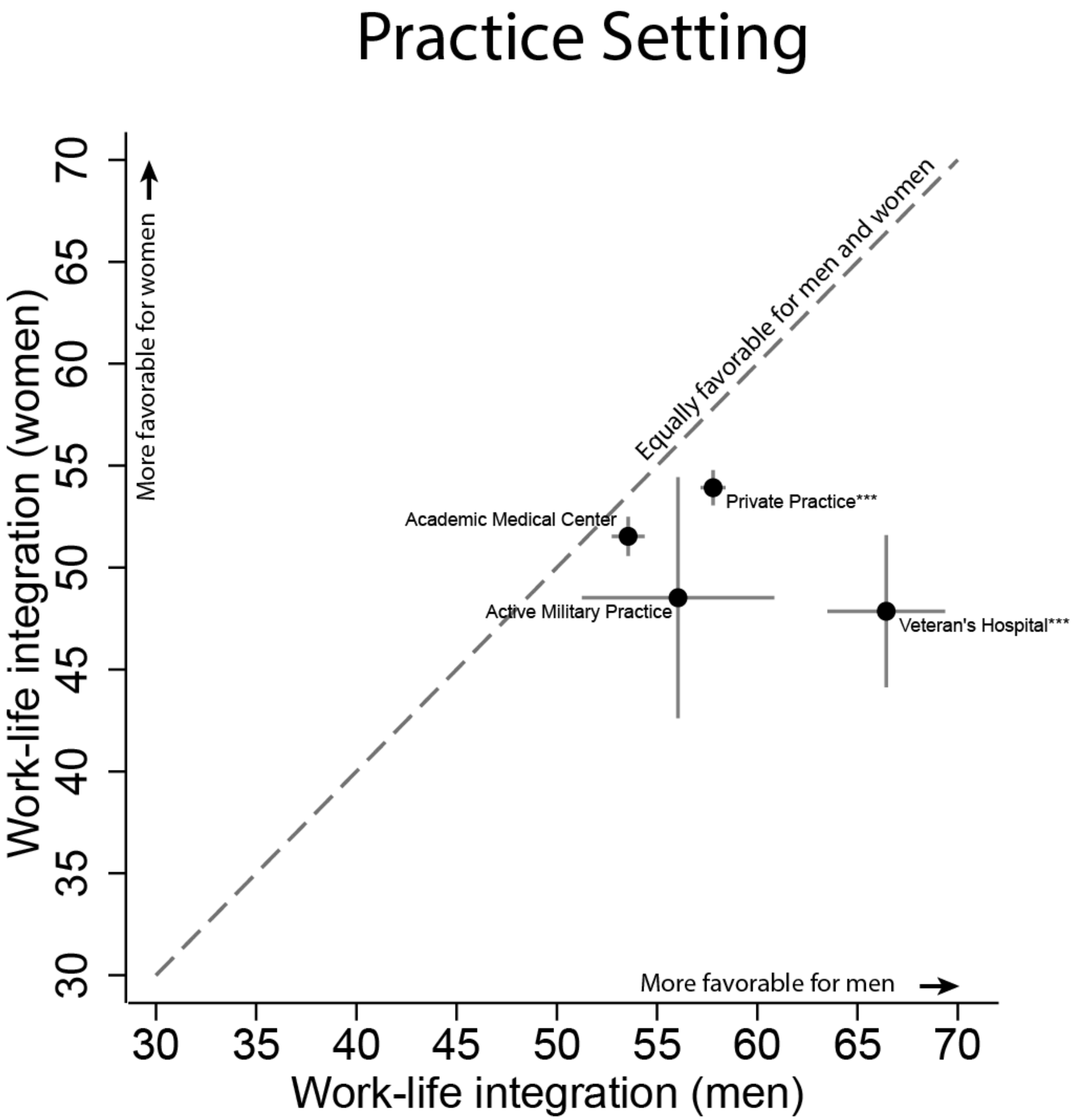

eFigure 4. Work-life integration by practice setting, for males (x-axis) and females (y-axis), shown as means and standard errors. Asterisks denote significantly different work-life integration scores between male and female respondents, via two-tailed t-test. * indicates $P < .05$; ** indicates $P < .01$; *** indicates $P < .001$

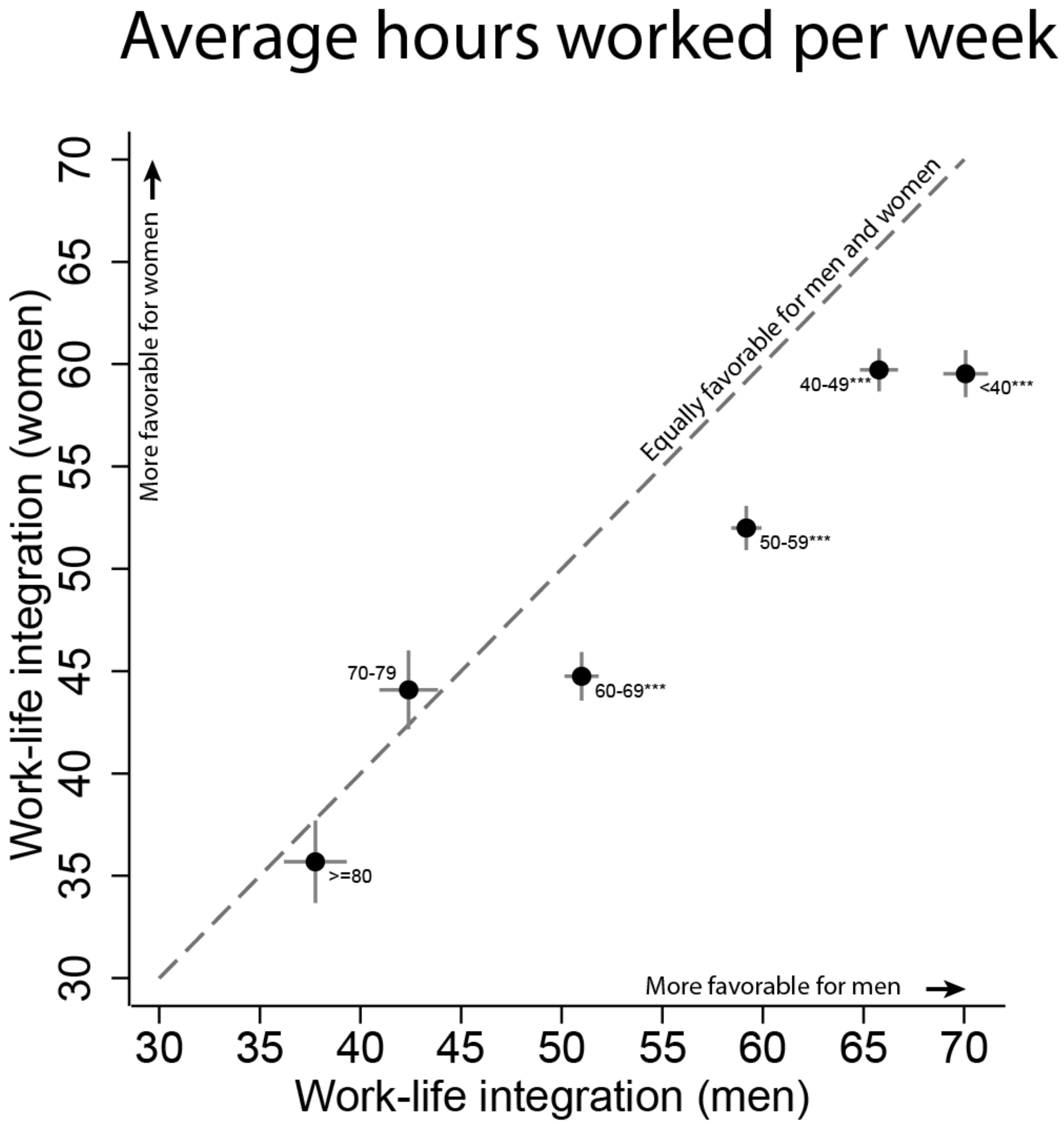

eFigure 5. Work-life integration by hours worked per week, for males (x-axis) and females (y-axis), shown as means and standard errors. Asterisks denote significantly different work-life integration scores between male and female respondents, via two-tailed t-test. * indicates $P < .05$; ** indicates $P < .01$; *** indicates $P < .001$

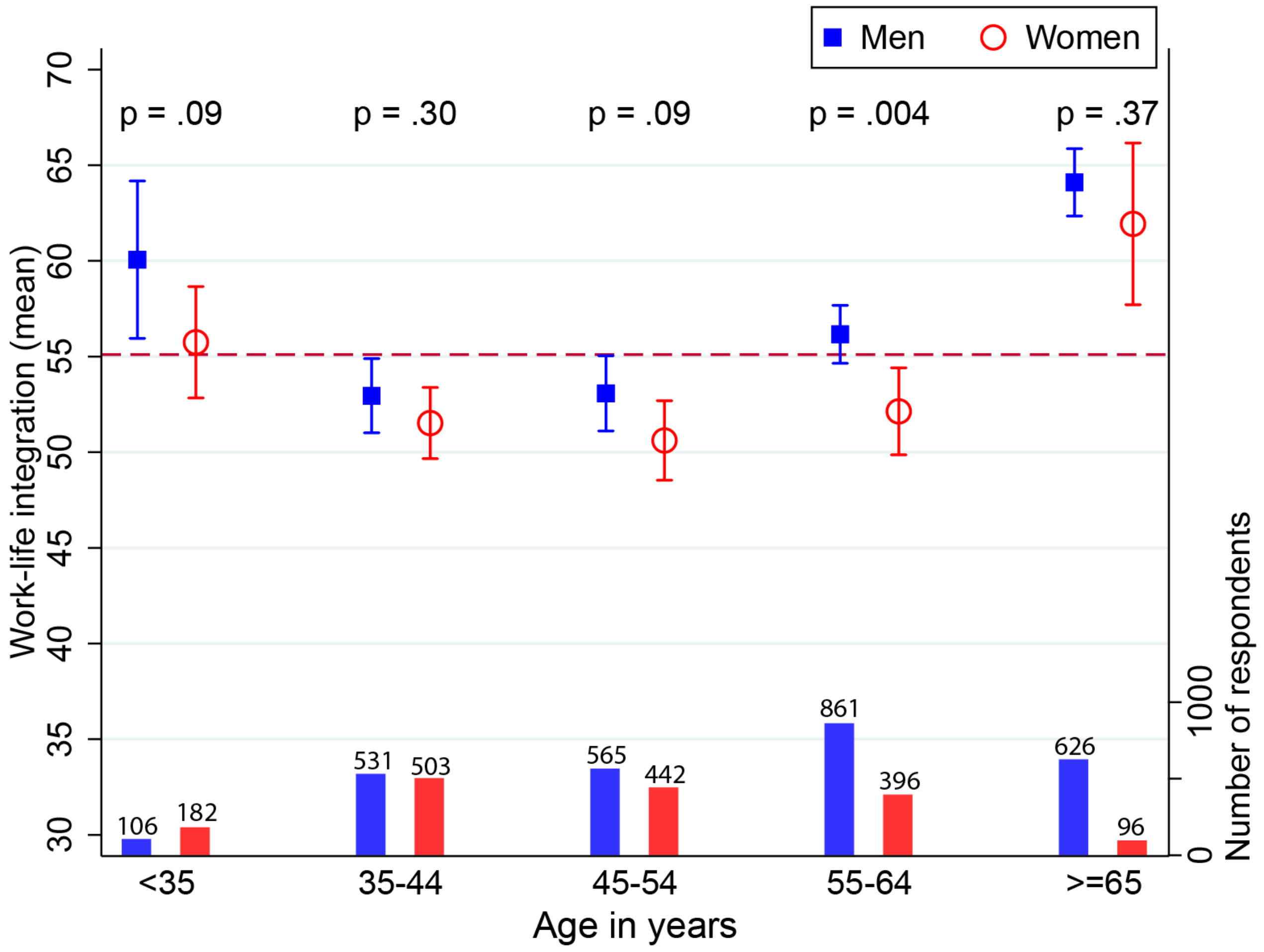

eFigure 6. Problems with work-life integration by physician sex and physician age. Data shown as means and 95% confidence intervals. P values obtained via two-tailed t-test. Number of respondents within each category shown for reference. Reference line at the population mean of 55.1.

© 2021 Tawfik DS et al. *JAMA Network Open.*

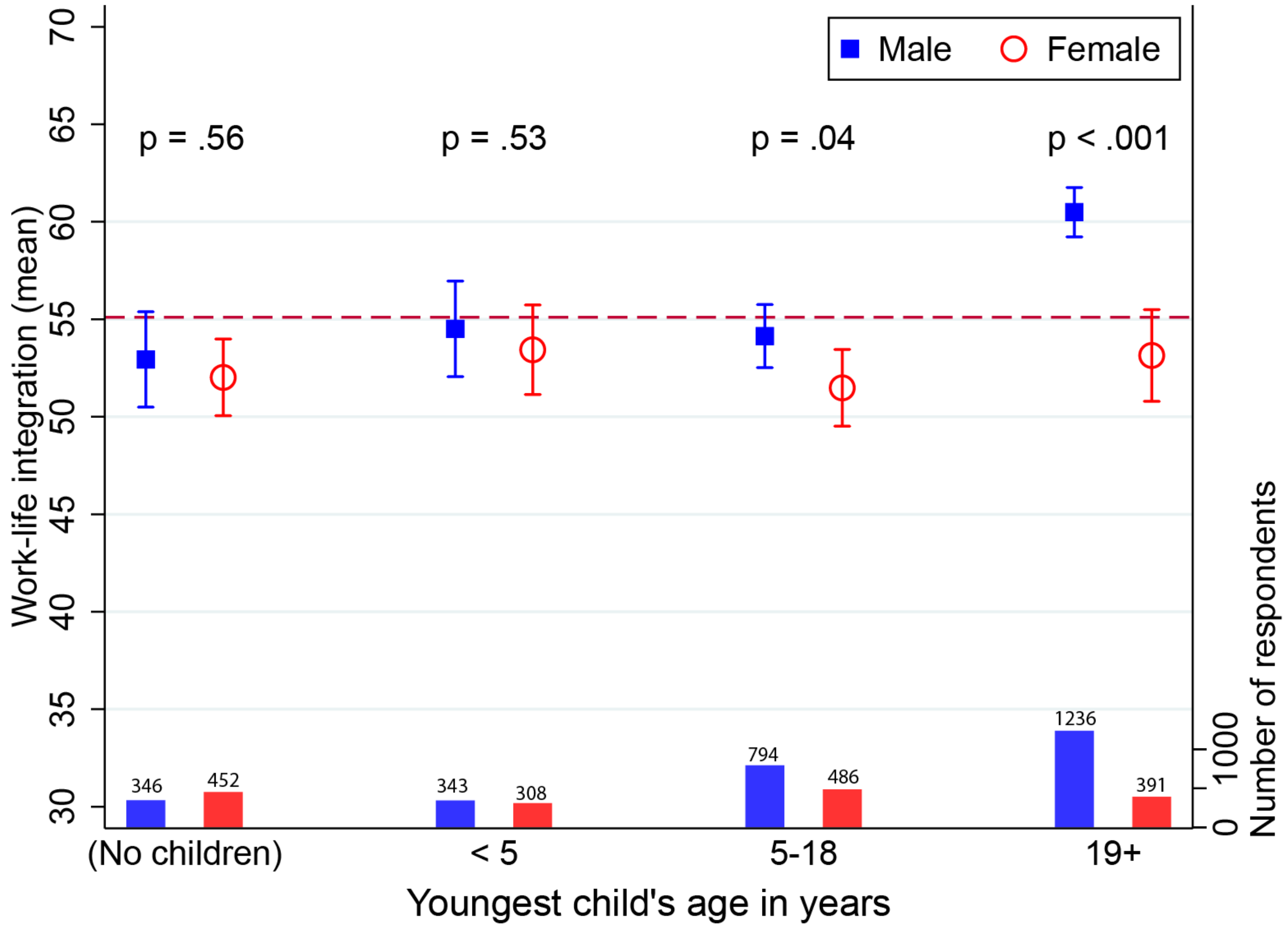

eFigure 7. Work-life integration by physician sex and youngest child's age in years. Data shown as means and 95% confidence intervals. P values obtained via two-tailed t-test. Number of respondents within each category shown for reference. Reference line at the population mean of 55.1.

© 2021 Tawfik DS et al. *JAMA Network Open.*

eTable 1. Univariate screen of variables associated with work-life integration.

	Coefficient	SE	*P*-value	Overall *P*-value*
**Specialty**				<.001
Internal Medicine Subspecialty	-1.3	3.9	.74	
General Internal Medicine	-0.9	3.9	.82	
Psychiatry	5.1	3.9	.20	
Family Medicine	-1.6	3.9	.67	
General Surgery Subspecialty	-8.0	3.9	.04	
Emergency Medicine	-10.2	4.0	.01	
Orthopedic Surgery	-0.7	4.0	.86	
General Pediatrics	5.8	4.0	.15	
Anesthesiology	-1.3	4.1	.75	
Pediatric Subspecialty	1.1	4.1	.78	
Radiology	1.3	4.1	.75	
Neurology	1.2	4.1	.76	
Obstetrics and Gynecology	-4.5	4.2	.28	
General Surgery	-8.1	4.2	.054	
Ophthalmology	7.6	4.2	.07	
Pathology	4.0	4.3	.35	
Dermatology	6.4	4.3	.13	
Physical Medicine and Rehabilitation	-1.1	4.3	.81	
Neurosurgery	-8.4	4.8	.08	
Radiation Oncology	-0.5	5.3	.92	
Otolaryngology	-2.3	5.3	.66	
Urology	-13.7	5.7	.02	
Preventive Medicine / Occupational Medicine	-0.0	6.0	.99	
Other	1.6	4.3	.71	
**Age (years)**				<.001
< 35	4.6	3.2	.16	
35-44	-0.6	3.0	.85	
45-54	-0.8	3.0	.79	
55-64	2.1	3.0	.49	
≥ 65	11.0	3.1	<.001	
**Years in Practice (per year)**	0.3	0.0	<.001	
**Nights on call per week (per night)**	-1.6	0.2	<.001	
**Hours worked per week**				<.001
< 40	16.0	2.6	<.001	
40-49	14.3	2.6	<.001	
50-59	7.7	2.6	.003	
60-69	0.08	2.6	.98	
70-79	-5.9	2.8	.03	
≥ 80	-11.9	2.8	<.001	
**Primary practice setting**				<.001
Private Practice	15.0	4.2	<.001	
Academic Medical Center	11.1	4.2	.009	
Veteran's Hospital	17.4	4.8	<.001	
Active Military Practice	11.8	5.3	.03	

© 2021 Tawfik DS et al. *JAMA Network Open.*

Not In Practice or Retired	19.1	4.8	<.001	
Other	13.2	4.2	.002	
**Sex**				<.001
Male	10.2	5.2	.052	
Female	5.7	5.2	.28	
Other	-9.2	8.9	.30	
**White/Caucasian**	0.9	0.9	.31	
**Asian**	0.3	1.1	.78	
**Black/African American**	-3.5	2.1	.10	
**American Indian/Alaskan Native**	2.2	5.1	.67	
**Pacific Islander/Native Hawaiian**	1.5	4.7	.76	
**Hispanic or Latino**	-1.0	1.5	.48	
**Other**	0.8	1.6	.63	
**Relationship status**				<.001
Married	9.4	4.9	.054	
Single	3.4	5.0	.50	
Partnered	6.5	5.1	.21	
Widow/Widower	11.0	5.8	.06	
**Age of youngest child**				<.001
(no children)	-8.0	1.0	<.001	
< 5 years	-6.2	1.1	<.001	
5-18 years	-7.1	0.9	<.001	
≥ 19 years	-6.4	1.3	<.001	

N = 4370 respondents. Dependent variable is work-life integration score (0-100 point scale) Estimates via separate univariate linear regressions. *Overall *P*-values for categorical variables via Wald test.

© 2021 Tawfik DS et al. *JAMA Network Open.*

eTable 2. Interaction model. Multivariable linear regression showing personal/professional factors and interactions as independent variables associated with work-life integration.

Variable	Coefficient	SE	P-value	Overall P-value*
Intercept	74.32	2.69	<.001	
Woman (vs. man)	-7.72	3.09	.01	
Age in years (vs. < 35 years)				.008
35-44	-7.53	2.24	.001	
45-54	-6.88	2.38	.004	
55-64	-5.92	2.42	.01	
≥ 65	-4.36	2.57	.09	
Sex-Age interaction				.04
Woman, 35-44 years	3.08	2.90	.29	
Woman, 45-54 years	1.15	3.13	.71	
Woman, 55-64 years	2.12	3.29	.52	
Woman, ≥ 65 years	9.47	3.98	.02	
Relationship status (vs. married)				.84
Single	-0.31	1.62	.85	
Partnered	0.28	2.30	.90	
Widow/Widower	-6.87	7.84	.38	
Youngest child's age (vs. no children)				.01
< 5 years	3.70	1.82	.04	
5-18 years	4.24	1.63	.009	
≥ 19 years	5.73	1.72	.001	
Sex-Youngest child's age interaction				.02
Woman, youngest child < 5 years	-3.97	2.36	.09	
Woman, youngest child 5-18 years	-5.02	2.09	.02	
Woman, youngest child ≥ 19 years	-7.00	2.41	.004	
Relationship status-Youngest child's age interaction				<.001
Single, youngest child < 5 years	-11.73	5.95	.049	
Single, youngest child 5-18 years	-7.92	2.76	.004	
Single, youngest child ≥ 19 years	-3.87	2.70	.15	
Partnered, youngest child < 5 years	-2.58	7.61	.74	
Partnered, youngest child 5-18 years	-11.46	4.59	.01	
Partnered, youngest child ≥ 19 years	-4.69	3.83	.22	
Widow/Widower, youngest child < 5 years	64.67	21.88	.003	
Widow/Widower, youngest child 5-18 years	18.23	9.83	.06	
Widow/Widower, youngest child ≥ 19 years	2.23	8.84	.80	
Specialty (vs. Internal Medicine Subspecialty)				<.001
Anesthesiology	-2.85	1.72	.10	
Dermatology	2.60	2.16	.23	
Emergency Medicine	-17.44	1.66	<.001	
Family Medicine	-3.50	1.46	.02	
Radiology	0.87	1.85	.64	
Neurology	0.07	1.88	.97	
Obstetrics and Gynecology	-1.57	1.91	.41	
Ophthalmology	2.90	2.08	.16	
Pathology	3.43	2.12	.10	
Physical Medicine and Rehabilitation	-2.35	2.24	.29	
Psychiatry	1.33	1.46	.36	
Other	-1.02	2.19	.64	
General Internal Medicine	-0.43	1.45	.76	

© 2021 Tawfik DS et al. *JAMA Network Open.*

General Pediatrics	2.50	1.70	.14	
Pediatric Subspecialty	2.23	1.79	.21	
General Surgery	-5.07	2.01	.01	
General Surgery Subspecialty	-3.69	1.48	.01	
Neurosurgery	0.92	3.07	.76	
Orthopedic Surgery	-0.72	1.65	.66	
Otolaryngology	-2.02	3.56	.57	
Urology	-10.07	4.02	.01	
Preventive Medicine/Occupational Medicine	-3.35	4.47	.45	
Radiation Oncology	-2.31	3.57	.52	
Hours worked per week (vs. < 40 hours)				<.001
40-49	-4.54	1.43	.001	
50-59	-12.28	1.37	<.001	
60-69	-19.84	1.40	<.001	
70-79	-28.07	1.80	<.001	
≥ 80	-32.79	1.90	<.001	
Sex-Hours interaction				<.001
Woman, 40-49 hours per week	3.58	2.08	.09	
Woman, 50-59 hours per week	3.18	2.05	.12	
Woman, 60-69 hours per week	3.93	2.15	.07	
Woman, 70-79 hours per week	12.17	2.87	<.001	
Woman, ≥ 80 hours per week	7.97	2.91	.006	

N = 4370 respondents. Dependent variable is work-life integration score (0-100 point scale) Estimates via multivariable linear regression with all covariates shown. *Overall *P*-values for categorical variables via Wald test.

© 2021 Tawfik DS et al. *JAMA Network Open.*
